# Kerosene in Surgery

**Published:** 1896-09

**Authors:** 


### SELECTIONS.

### Kerosene in Surgery.

A. Shirman, has observed that in treating wounds and ulcers of the trunk and of the limbs, among the poorer classes, by the usual antiseptic methods, recovery usually progresses very slowly on account of the fact that time and circumstances do not allow the patient to apply these preparations as often as necessary.

For this reason the author determined to try some other substance as an antiseptic, and it occurred to him to try kerosene in these cases.

For this purpose, in cases of ulcers, especially atonic and indolent ones, he smeared them with commercial kerosene, either pure or diluted (from 33 to 50 percent) with alcohol, with a small camel's hair brush or with a piece of gauze soaked in the solution. Shortly after the application a burning sensation was felt, but it soon passed away.

The appearance and character of the ulcers showed a change for the better; the discharge gradually diminished, and in the course of from two to four weeks the rapidly granulating surface formed a scar without any contraction of the surrounding parts.

The advantages of the use of kerosene for such cases, Dr. Shirman summarizes as follows: It produces healing in a comparatively brief space of time; it is much more economical and is easily obtained; it does not produce constitutional poisoning through the wound by absorption as other antiseptics sometimes do; it has not the intolerable smell of some of the others which are now in use; and the formation of a cicatrix on the ulcers proceeds rapidly. The author has never found the wound to be complicated with any erysipelatous process. Kerosene having a local irritating action on the wound undoubtedly possesses also disinfecting properties for the remote surface as well as for the adjacent surface around the wound. This is of great value, for actual facts show that persons residing in the kerosene oil districts are protected against ailments of an epidemic character—such as cholera, etc.—*N. Y. Med. Journal.*

